# Spontaneous Reorientation Is Guided by Perceived Surface Distance, Not by Image Matching Or Comparison

**DOI:** 10.1371/journal.pone.0051373

**Published:** 2012-12-12

**Authors:** Sang Ah Lee, Nathan Winkler-Rhoades, Elizabeth S. Spelke

**Affiliations:** 1 Center for Mind/Brain Sciences, University of Trento, Rovereto, Italy; 2 Department of Psychology, Harvard University, Cambridge, Massachusetts, United States of America; 3 Department of Brain and Cognitive Sciences, Massachusetts Institute of Technology, Cambridge, Massachusetts, United States of America; The Australian National University, Australia

## Abstract

Humans and animals recover their sense of position and orientation using properties of the surface layout, but the processes underlying this ability are disputed. Although behavioral and neurophysiological experiments on animals long have suggested that reorientation depends on representations of surface distance, recent experiments on young children join experimental studies and computational models of animal navigation to suggest that reorientation depends either on processing of any continuous perceptual variables or on matching of 2D, depthless images of the landscape. We tested the surface distance hypothesis against these alternatives through studies of children, using environments whose 3D shape and 2D image properties were arranged to enhance or cancel impressions of depth. In the absence of training, children reoriented by subtle differences in perceived surface distance under conditions that challenge current models of 2D-image matching or comparison processes. We provide evidence that children’s spontaneous navigation depends on representations of 3D layout geometry.

## Introduction

All animals, including humans, must keep track of their place within the navigable environment. Behavioral and neurophysiological research has long suggested that the sense of place depends on representations of the geometric structure of the environment [1], [2]. Early evidence for this proposal, from behavioral studies of maze learning in rats [3], was later disputed [4], [5], but compelling evidence for geometry-guided navigation came from studies of reorientation [6], [7]. Hungry rats who were disoriented after seeing food in a rectangular chamber subsequently dug for food only at the two locations specified by the shape of the chamber. While the rats learned over reinforced trials to use featural cues (such as distinctive odors, patterns, or a single light-colored wall), their initial disoriented searches depended only on the rectangular geometry of the environment. Evidence for this geometric environmental representation was later extended to animals of other species and ages, including ants [8] and newly hatched birds and fish [9], [10], [11]. Studies of both human adults [12], [13] and children [14] suggest that navigation primarily depends on the computation of the distance relationships and directions between extended surfaces rather than on local geometric properties such as surface lengths or corner angles.

Neurophysiological studies of oriented animals provide further evidence that navigation is guided by surface layout geometry. When oriented rats or humans move through a real or virtual arena, neurons such as the “boundary vector cells” have been found in the hippocampal formation that are activated automatically in relation to extended surface distances and directions, and not by landmark objects or by surface colors and patterns [15], [16], [17]. All these findings suggest that navigation depends on phylogenetically ancient, early developing processes sensitive to the environmental 3D surface layout.

Nevertheless, other findings raise problems for this view [18]. First, oriented insects and birds recognize particular, significant locations in an array, such as the location of nectar or buried food, by means of local, parallel processes for matching brightness contours in 2D panoramic images of the array [5]. Elegant experiments reveal that these recognition processes do not depend on surface distance: Animals who have learned to locate food within a configuration of small, nearby landmarks will generalize to a configuration of larger, more distant landmarks if the 2D image properties of the two arrays are the same [19]. Moreover, disoriented rats and humans can incorporate features such as 2D patterns or color contours into their spatial search after training or instruction (e.g., [10], [20], [21], [22]). Consistent with these findings, neurons encoding the location and heading direction of oriented rats sometimes are anchored to such contours (e.g., [23], [24], [25]) and alter their response patterns markedly if the environment changes in coloring or shape (e.g., [26]).

These findings have motivated two alternative hypotheses concerning the representations guiding navigation. One proposal appeals to processes for matching stored 2D images of the environment to images perceived during navigation [18]. Recent computational models show that image-matching processes can account for the primary findings from behavioral studies of reorientation [27], [28], [29], [30] and neurophysiological studies of oriented navigation [29] in non-human animals. Image-matching theories also can explain several findings from studies of children: When children are disoriented in a square room whose alternating walls differ in brightness [31], [32], they can match the stored image of the goal location in accord with these brightness relations, even though they fail to use such relations in a rectangular room with a single wall of contrasting brightness. In a rectangular room with one wall of a distinctive color or brightness [33], the salience of the discrepancy between visual images of longer and shorter walls may be greater than the discrepancy between images of different colored walls, resulting in behavior primarily in accord with wall length rather than wall color. Image matching theories therefore account for reorientation in geometrically structured environments without representations of 3D properties such as surface distance.

**Figure 1:**
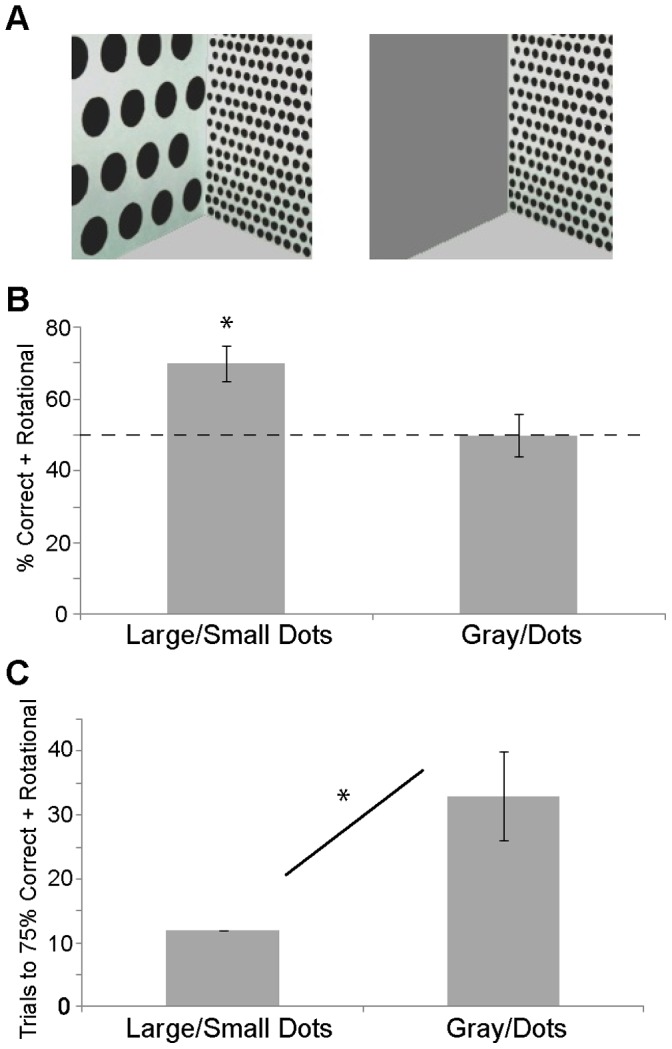
Sample testing spaces and results from [**32**] and [**39**]. A: Depiction of corner views of two of the square arenas tested with human toddlers [32] and mice [39]; B: Percentages of searches at the correct and rotational (the diagonal corner that is featurally/geometrically identical to the correct one) corners for Experiments 1 and 3 in [32] (asterisk indicates above-chance (50%) search); C: Number of trials required to meet a criterion of 75% searches at the correct and rotational corners for mice trained in [39] (asterisk indicates a significant difference between conditions). Data are replotted with permission from Stella Lourenco and Alexandra Twyman.

Nevertheless, other findings from studies of children are difficult to reconcile with image-matching theories. Children reorient spontaneously by subtle perturbations in the 3D surface layout, including a rectangular frame 2-cm-high and a speed-bump-like hill 10-cm-high, but not by more dramatic brightness contrasts in 2D forms or object arrays [34], [35]. Children also reorient by distance differences between surfaces of equal length, but not by length differences between surfaces at equal distances [14], despite the similar image properties of these arrays. Finally, children reorient in square environments whose alternating walls contrast in pattern size and density, but not in square environments whose alternating walls contrast in pattern presence or absence ([31], [32], [36]; Figure 1a and 1b). These findings, replicated in chicks [37] and fish [38] in studies of spontaneous reorientation and in mice [39] in studies comparing learning rates in various environments (Figure 1c), have motivated a second alternative to reorientation mechanisms attuned to distance relationships.

According to Huttenlocher and Lourenco [36], humans and animals assign directional relationships to any continuous perceptual variables, whether these variables are captured only by 3D surface representations (e.g., wall distance) or by 2D images as well (e.g., wall brightness). Humans and animals reorient by matching the current directional variables to those that were experienced prior to disorientation. On this view, children’s and animals’ failures to reorient by differences in color or pattern, or by the presence vs. absence of landmark objects or patterns [32], [36], stem from the discontinuous character of these features in the tested environments. Because this theory assigns no special status to spatial variables, it also challenges the hypothesis of a specific process for navigating by representations of 3D layout geometry.

Although theories of reorientation by 2D-image matching and by relational comparison have received wide attention, neither has been tested directly against the competing hypothesis of reorientation by surface distance. The evidence that animals and children reorient in square environments whose walls differ in brightness or pattern size and density could be explained not only by image matching and relational comparison but also by representations of surface distances, because these stimulus manipulations can induce illusions of depth. Surfaces differing in brightness may be perceived as differing in distance or orientation, in accord with the depth cue of *shading*: brighter surfaces tend to appear closer to the light source or oriented more nearly perpendicular to its direction [40]. Moreover, surfaces containing elements of the same shape at different scales may be perceived as differing in distance from the observer, in accord with the depth cues of *relative size* and *texture density*: surfaces containing larger, sparser elements appear closer to the observer [41], [42].

If surfaces differing in brightness or in pattern size and density influence navigating animals’ perception of surface distance, then theories of geometry-guided navigation could account for the evidence to which the rival theories appeal. When a child or animal stands in the center of a square arena with internal light sources, walls with greater brightness or larger, sparser patterns will appear closer than those that are darker or more densely patterned, leading to the perception of a slightly rectangular arena. Navigators might use this perceived asymmetry in distance to reorient themselves [6].

Here we test these competing theories by following the logic of a century of experiments on depth perception. Behavioral responses to any single depth cue are ambiguous: They could depend either on representations of relationships within the 2D sensory image or on representations of distance in the 3D layout. Distance, however, is specified by multiple cues. If behavioral responses depend on perceived distance, then these cues should interact: When two cues are arranged to specify that one of the surfaces is closer than the other, then perception of the differing depths of the two surfaces should be enhanced; if the same two cues are arranged to specify opposite distance relationships between the surfaces, then perception of the differing depths of the two surfaces should be diminished [43].

To test both depthless image matching and relational comparison theories against theories postulating a process of reorientation only by surface geometry, therefore, we investigated children’s reorientation in arenas whose walls differed both in actual distance and in either surface brightness or pattern size and density. Because pictorial cues to depth evoke perceptions of only small differences in distance when they are placed in competition with other cues, we tested for interactions between pictorial and other depth cues by using subtly rectangular rooms, and we conducted this test in three steps.

**Figure 2:**
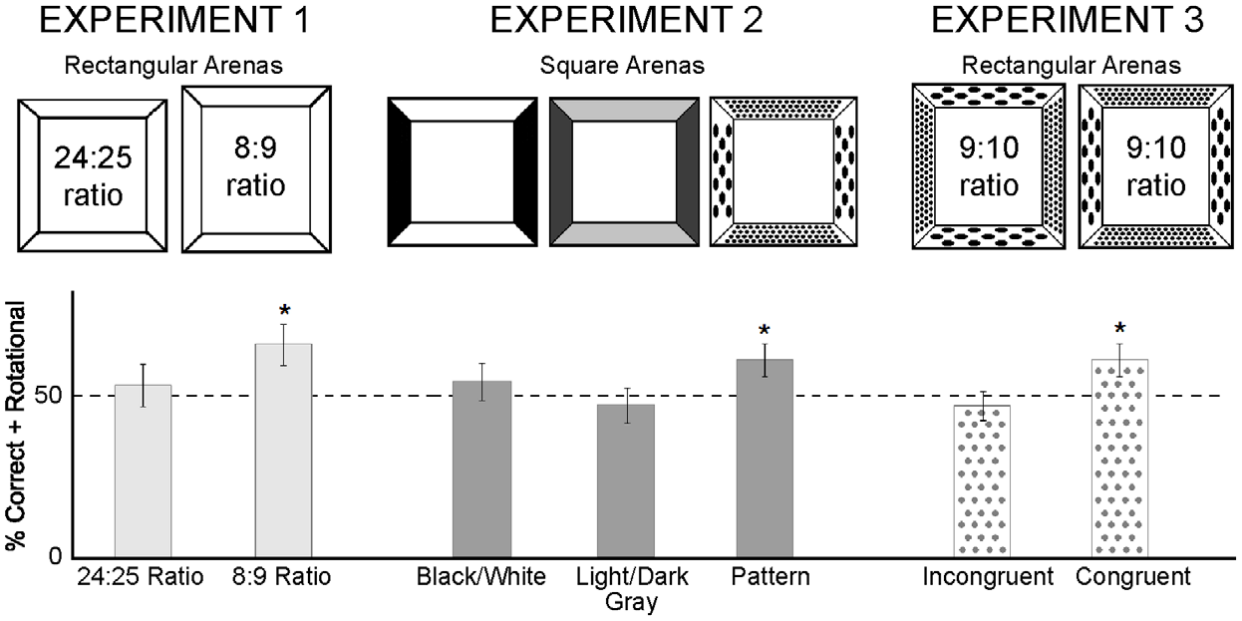
Search results for each experiment. Arenas tested in Experiments 1 (left), 2 (center), and 3 (right) and the percentages of searches in the correct and rotational corners in each arena (bottom). Asterisks indicate above-chance (50%) search.

In Experiment 1, we investigated 3-year-old children’s reorientation in homogenous, subtly rectangular enclosures, in order to estimate the minimal aspect ratio at which children reorient by this shape. Following the method of Lee & Spelke [35], children were introduced into a rectangular arena placed at the center of a fully symmetrical cylindrical room. After an object was hidden in one corner of the enclosure, children turned with eyes closed until they were disoriented and then were encouraged to find the object. If children reoriented by the enclosure’s shape, they should confine their search to the two geometrically specified corners. Children were found to be strikingly sensitive to small differences in surface distance: They reoriented by the shape of a rectangular arena whose sides differed in distance by a ratio of 8∶9 (Figure 2, left).

Next we attempted to reproduce past findings that children reorient, in a square environment, by differences in surface brightness or pattern size and density [31], [32], [36]. Our first attempt to replicate these findings used children (n = 32) of the age of those in Experiment 1, tested by the same methods as in that experiment. The findings were entirely negative: children searched randomly at the four corners of the square enclosure, both when its alternating walls differed in brightness (46.1% search at the two corners specified by the brightness cue, chance = 50%, t(15) <1, n.s.) and when its alternating walls differed in pattern size and density (50.1% search at the two corners specified by the relative size cue, chance = 50%, t(15) <1, n.s.). Although the method of Experiment 1 provided evidence for reorientation by subtle differences in surface distance, our use of this method failed to replicate past findings of reorientation by large differences in surface brightness or pattern size [31], [32], [36].

**Figure 3:**
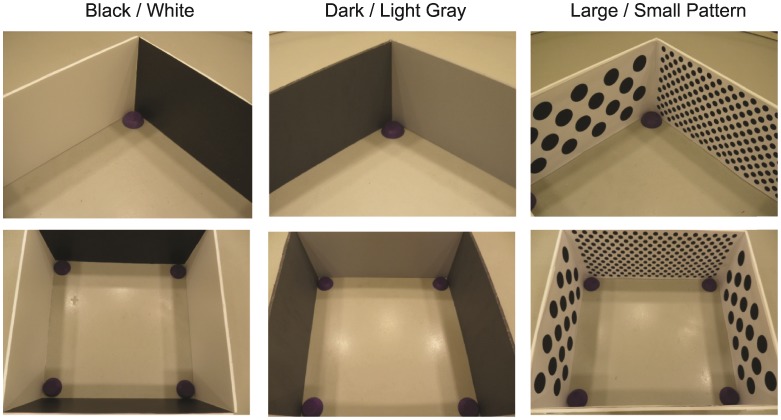
Displays for Experiment 2. Perspective and overhead views of the black/white, dark/light gray, and patterned arenas tested in Experiment 2.

Because Experiment 1 used a different age range, design, and procedure from those of the published studies on which it was based, we shifted our methods in the next experiment so as to follow closely those used by past investigators who reported both the brightness effect and the pattern size effect [32], [36]. In Experiment 2, we tested 18- to 24-month-old children in a small square arena with alternating walls that respectively were black and white [31], dark and light gray [32], or patterned with circles that were large and sparse or small and dense [36] (Figure 3). This experiment failed to replicate either of the two published brightness effects, but it successfully replicated the effect of pattern size and density (Figure 2, center).

Finally, in Experiment 3, we tested for interactions between the effects of surface distance and pattern size by investigating 18-24-month-old children’s reorientation within a slightly rectangular room whose pairs of opposite walls were covered by circles that were either large and sparse or small and dense. The four-year-old children in Experiment 1 successfully reoriented in a rectangular room with an aspect ratio of 8∶9 (0.889) but not 24∶25 (0.96), suggesting that intermediate aspect ratios would be near threshold at this age. Because the children to be tested in Experiment 3 were somewhat younger, we tested them in a room with an aspect ratio that was only slightly less elongated than the detectable ratio from Experiment 1∶9:10 (0.90). Because children in Experiment 2 successfully reoriented in a square room covered with the patterns of different sizes and spacing [36], Experiment 3 used those patterns in two conditions in which the larger, sparser circles appeared on the walls that were either closer to or farther from the center of the chamber (Figure 2, right).

If relative size influences children’s reorientation directly and independently of any effect on perceived surface distance, then children should reorient successfully in both conditions. In contrast, if relative size influences children’s reorientation by serving as a depth cue, then this cue should interact with other depth cues, such as binocular disparity and motion parallax, that indicate the true distances of the surfaces. When large circles are placed on the surfaces that are slightly closer to the child, reorientation by layout geometry should be enhanced. When the same circles are placed on the surfaces that are slightly more distant, reorientation should be diminished.

## Materials and Methods

### Ethics Statement

Informed consent was obtained in writing from the guardians on behalf of the young participants, and verbal consent was obtained from the children. Either the guardians or participants could choose to stop the experiment at any time. All experiments and consent procedures were approved by the Institutional Review Board at Harvard University for research on human subjects.

### Experiment 1

#### Participants

16 children (8 boys), aged 35 to 42 months (M = 39 months), were tested. Four additional children failed to complete the experiment. In all of the experiments, children were tested only in one experiment, and they were naïve to the experimental arena at trial 1.

#### Apparatus

Testing occurred within a circular, 3.66-m diameter room consisting of twelve curved wall panels (one of which was a spring-operated door that was indistinguishable from the other eleven panels from the inside of the testing space), soundproof walls, a solid light floor, and six circular lights arranged symmetrically around a circular fish-eye-lens camera mounted at the center of the 2.34-meter-high ceiling. At the room’s center was a rectangular enclosure composed of 1.02-meter-high white walls. One of the shorter walls served as the door (from inside the enclosure it was indistinguishable from the opposite wall) and was movable between two locations to create enclosures of 1.22 m by 1.37 m (a length ratio of 8∶9), or 1.22 m by 1.27 m (a length ratio of 24∶25). The corners were covered with 5-cm-wide panels, oriented 45° to both walls, behind which a sticker could be hidden.

#### Design

Each child performed four trials in each arrangement of the enclosure, in a block design with array order counterbalanced across subjects. The hiding location was held constant across all trials for a given child but was counterbalanced across children. Children faced a different wall on each trial of each condition; the order of the four facing directions was counterbalanced across children.

#### Procedure

A child entered the room with an experimenter while parents remained outside and observed the study on a video monitor. The experimenter then fixed the movable wall to one of the two distance settings. In all the experiments, children were motivated to search after disorientation through a hiding and finding game. First, the child chose a sticker and watched as the experimenter placed it behind one corner panel. Then the child was blindfolded and turned in place until disoriented (typically 3–4 rotations). Disorientation was checked by asking the child to point to the door while blindfolded; turning resumed if the child pointed correctly. After disorientation was confirmed, the experimenter stood behind the child, faced the child towards one of four predetermined directions, removed the blindfold, and encouraged the child to find the sticker. Once children made their first choice by reaching into the hiding location, the experimenter stopped the trial by preventing subsequent search attempts, retrieving the sticker, and moving onto the next trial. After the first block of trials, the movable wall was removed for the child to exit, and he/she was taken out of the room briefly. Upon returning, the experimenter attached the movable wall at the second distance setting before starting the second condition. The location of the first search (coded as the first corner flap lifted) was recorded from the video record.

### Experiment 2

#### Participants

Forty-eight children (24 girls), aged 18 to 24 months (M = 21 months) took part in the experiment. Six additional children failed to follow directions (e.g., cover his/her eyes) or to complete the experiment.

#### Apparatus

Children were tested in the same cylindrical room as in Experiment 1, furnished with a centrally placed, 97 cm by 97 cm square enclosure with contrasting pairs of opposite walls (see Figure 3). In the black/white condition, alternating walls were covered with black or white contact paper. In the gray condition, the walls were painted dark or light gray, matching samples provided by Lourenco et al. [32]. In the pattern condition, the walls were painted white and covered with black circles that were either 8.9 cm or 2.5 cm in diameter, spaced so as to equate their average brightness (by presenting the same total area of black dots on each wall) and to scale item density to item size (see Figure 2). Inverted opaque bowls at each corner served as the hiding locations.

#### Design

Each child was tested in one condition. As in Experiment 1, children performed 4 reorientation trials with a single hiding place and four different facing directions; both hiding place and order of facing directions were counterbalanced across the children in each of the three versions of this experiment.

#### Procedure

In contrast to Exp. 1, and following the procedure of past research with these displays [32], [36], the parent was present in the room and testing was performed by the experimenter and the parent together. While the experimenter stood outside of the enclosure, the parent picked up the child and stepped into the center of the enclosure. The experimenter called attention to the walls of the arena and then showed the child a small toy and made sure that the child attended to it as she placed it under one of the bowls. Then the parent picked up the child, covered or shaded the child’s eyes such that the child could not look down and track the location, and rotated in place 3–4 times. Meanwhile, the experimenter walked around the box while reminding the child to keep his or her eyes covered or closed. After the child was faced toward one wall and released, the parent stepped out of the box and stood next to the experimenter, who stood on the other side of the wall that the child faced. If the child expressed a desire for the parent to stay inside the box, the parent was instructed to stand quietly behind the child, with his/her gaze fixed directly ahead on the floor or into the child’s eyes (if the child looked up at the parent), until the child searched. The child was encouraged to find the toy. After the first search, coded by his/her lifting of one of the corner hiding containers, the experimenter retrieved the toy and moved on to the next trial.

### Experiment 3

#### Subjects

Participants were 32 children aged 18–24 months (8 boys and 8 girls in each condition; mean age 21 months). Three additional children failed to cooperate or to complete the task.

#### Apparatus

The apparatus was the same as in the Pattern condition of Experiment 2 except for the lengths of the walls (92 cm and 102 cm), resulting in a subtly rectangular box. In the Congruent condition, the larger circles appeared on the walls that were closer to the center of the box. In the Incongruent condition, the larger circles appeared on the walls that were more distant from the center of the box.

#### Design and procedure

Children were tested following the procedures of Experiment 2. Separate groups of children (n = 16 per group) were tested in the Congruent and Incongruent conditions. Within each condition, the hiding location was counterbalanced across children, the child’s facing direction was counterbalanced across trials, and the order of different facing directions was counterbalanced across children.

## Results

### Experiment 1

Figure 2 (left) presents the principal findings. Preliminary analyses revealed no effects of enclosure order or participant sex (F-values<1, n.s.), so further analyses collapsed across these factors. Three-year-old children searched equally at the correct and opposite corners in both the 8∶9 and 24∶25 rectangular enclosures (for both enclosures, t(15)<1, n.s.), showing that they were disoriented. Moreover, children searched the geometrically correct corners of the 8∶9 enclosure on 66% (S.E. = 6.4) of trials (chance = 50%, t(15) = 2.44, p<0.05), providing evidence that they reoriented by this difference in distance between walls. In contrast, children searched randomly in the 24∶25 enclosure, searching geometrically correct corners on 53% (S.E. = 6.4) of trials (t(15)<1, n.s.). Nevertheless, children’s combined performance across these two conditions rose reliably above chance, t(15) = 2.24, p<0.05, and performance in the two enclosures did not differ reliably, (t(15) = 1.29, n.s.).

Experiment 1 provides evidence that children reorient in a rectangular enclosure whose walls differed in distance by only 11%. Thus, children reorient not only in rectangular environments whose aspect ratio is highly distinctive but also in those whose elongation is quite subtle. Together with other recent findings [14], [35], this finding adds to the evidence for a robust effect of surface distance on reorientation.

This finding raises the possibility that relative pattern size or shading influences reorientation by altering children’s perception of surface distances. Although relative size and surface brightness would be expected to change the perceived distances of the walls of a square room only slightly, such a perturbation might guide children’s reorientation if they perceive such a room as slightly rectangular. Before testing this possibility, however, we first attempted to replicate the brightness and pattern size effects obtained in previous experiments with square rooms, by testing 18–24 month old children in square rooms whose alternating walls were (a) black and white, (b) dark and light gray, or (c) patterned with elements that were large and sparse or small and dense (Figure 3).

### Experiment 2

Figure 2 (center) presents the primary findings. Because there were no sex differences in any of the three conditions (in all three conditions, t(14)<1), all analyses collapsed across gender. In each of the three conditions, children searched equally in the correct and the opposite rotationally symmetrical corners, providing evidence that they were disoriented (Black/White Condition, 25% vs. 30% search; Gray Condition, 23% vs. 23%; Pattern Condition, 30% vs. 31%; in all three conditions, t(15)<1). The primary analyses therefore compared search at the two corners with the correct brightness or pattern relationships to the two incorrect corners.

In the Black/White Condition, children searched in the two correct corners on 55% (S.E. = 6.1) of trials (chance = 50%, t(15)<1, n.s.), providing no evidence that children reoriented by using the black and white brightness difference (Figure 2, center). In the Gray condition, children searched in the two correct corners on 47% (S.E. = 5.5) of trials (t(15)<1, n.s.), also providing no evidence for reorientation using the brightness differences between the gray walls. In the Pattern Condition, children searched in the two correct corners on 61% of trials (S.E. = 5.1), t(15) = 2.15, p<0.05. Children therefore used the difference in pattern size and density to reorient themselves.

Experiment 2 failed to replicate the brightness difference effect reported by previous investigators [31], [32], despite the use of the same lightness values as in each of those experiments. It is possible that the brightness effect depends on conditions of illumination that we failed to recapture in the present studies; we return to this possibility in the Discussion.

More positively, Experiment 2 successfully replicated spontaneous reorientation in square environments with small/dense and large/sparse wall patterns reported in past studies of young children [36] and produced findings in accord with the faster goal learning in such environments shown by mice [39]. Accordingly, Experiment 3 tested two different interpretations of this effect by investigating the search patterns of children who were disoriented within a slightly rectangular room whose walls displayed the same patterns.

### Experiment 3

The principal findings appear in Figure 2 (right). Because there were no sex differences (both ts(14)<1, n.s.), all analyses collapsed across males and females. Children aged 18–24 months searched equally at the correct and opposite corners of the room in both enclosures (Congruent condition, 33% vs. 28%; Incongruent condition, 23% vs. 23%; in both conditions t(15)<1, n.s.), showing that they were disoriented. The primary analyses therefore compared search at the two corners with the correct pattern relationships to the two incorrect corners (Figure 2, right).

In the Congruent condition, children searched at the two correct corners on 61% (S.E. = 5.1) of trials, (chance = 50%, t(15) = 2.41, p<0.05). In the Incongruent condition, in contrast, children searched the correct corners only on 47% (S.E. = 4.5) of trials (t(15)<1). Performance in the two conditions differed reliably (t(30) = 2.07, p<0.05).

In the condition in which larger, sparser dots appeared on the closer sides of the enclosure, children reliably searched the corners with the appropriate directional relationship to the larger dots, as they did in the square room in Experiment 2 and in Huttenlocher and Lourenco’s [36] original experiment. In contrast to the predictions of the relational processing account, however, children failed to search the corners with the appropriate directional relationship to the larger dots when the larger, sparser dots appeared on the more distant walls.

Comparing across conditions, the placement of the dot patterns interacted with the direction of rectangularity of the arena. This finding provides evidence that the patterning cue served as a depth cue for children, as it does for younger infants [41] and adults [42], [43], leading them to perceive an objectively square space as slightly rectangular. When the large, sparse circles appeared on the closer walls, such that the cues of relative size and texture density were congruent with other depth information, children successfully reoriented. Their reorientation was impaired, however, when the small, dense circles appeared on the closer walls, such that the relative size depth cues conflicted with other cues to surface distance.

Could processes of depth perception also account for children’s reorientation in rooms whose alternating walls differ in brightness? We investigated this possibility by testing a new group of 18-24-month-old children (n = 32) using the same method as Experiment 3, in slightly rectangular rooms with alternating black and white walls. Again, we failed to replicate the brightness effect reported in other laboratories [31], [32]. Children searched the two corners with the correct brightness relationship no more than those with the incorrect brightness relationship, both in the Congruent condition in which the brighter walls were closer (56% search at the correct corners, chance = 50%, t(15) = 1.00, n.s.) and in the Incongruent condition in which the darker walls were closer (53% search at the correct corner, chance = 50%, t(15)<1, n.s.). Once again, we found no evidence that young children reorient by brightness differences between surfaces in the surrounding layout.

## Discussion

The present findings provide evidence that children’s reorientation depends on an analysis of surface distances and directions: Two fundamental aspects of 3D layout geometry. Although Experiment 2 replicated the finding [36] that children reorient in a square room whose alternating walls present the same pattern at two different scales, Experiment 3 indicated that this patterning influenced children’s perception of the relative distances of the adjacent surfaces at each corner. This finding accords with a century of research providing evidence that pattern size and density serve as depth cues [42], [44], beginning in infancy [41]. It also can account for the finding that children and mice respond to a difference in pattern size and density more readily than to what should otherwise be a more salient difference in pattern presence vs. absence (see Figure 1, bottom). In all these studies, reorientation may depend on the perceived distances and directions of the bounding surfaces of the enclosure.

Although our findings reveal a navigational process that depends on representations of surface distances, our findings do not reveal what reference frame children use to encode these relationships. Children might encode surface distance relative to the self: The distance of each surface from their position at the center of the array. Alternatively, children might encode the distances of each surface relative to the opposite or adjacent surfaces. Further research is needed to address this question.

The present findings provide the first evidence that children reorient by differences in surface distance not only when those differences are large, as in the highly elongated rectangular environments used in past experiments, but also when they are quite subtle. In Experiment 1, children reoriented by the distances and directions of surfaces that differed in distance by a ratio of only 8∶9. In Experiments 2 and 3, they reoriented by the depth cue of relative size, even though that cue induces only subtle perceptions of relative distance. These findings join recent evidence for reorientation by very small 3D surface perturbations [35] to provide evidence that navigating children are highly sensitive to 3D layout geometry.

The finding that children reorient by 3D layout geometry does not preclude the possibility that children also can learn to navigate by 2D-image matching or by non-spatial relational comparison. Indeed, multiple processes underlie children’s navigation, as evidenced by their use of the direct features of goal locations (i.e., the colors of containers or corners) to limit their searches to those locations. Nevertheless, our findings provide some evidence against both relational comparison theories and existing image matching theories of unreinforced spontaneous reorientation, at least as these theories apply to children. First, the findings of the Incongruent condition of Experiment 3 provide evidence against the hypothesis that children reorient by assigning directions to any detectable stimulus continuum. In contrast to the findings of Huttenlocher and Lourenco [36] and Lourenco et al., [32], the children in this condition failed to reorient by the difference in scale between the patterning on the alternating walls of the chamber. Although future research may reveal stimulus conditions in which children reorient by stimulus continua that do not influence perceived surface distance, the present findings suggest that a difference in pattern size and density, by itself, is not sufficient to guide children’s reorientation when it is presented under conditions that cancel the impression of depth that such patterns create.

The interaction between the geometric properties of the wall layout and dot patterns found in Experiment 3 also provides the clearest evidence to date against the predominant image matching theories that root reorientation in the processing of depthless “snapshots” of visual displays, and they cast doubt on any theory that would explain children’s navigation behavior exclusively on the basis of processes involving no representation of depth. If successful use of the dot patterns was achieved in Experiment 2 by 2D-image matching in the square arena, children should have applied the same process in Experiment 3, whose arrays differed from those of Experiment 2 only in depth. In contrast to the predictions of current image matching accounts (e.g., [18], [29]), the interaction of the differing cues to depth suggests that the processes guiding reorientation do not apply directly to static 2D images but are consistent with representations of surface distance. Nevertheless, the detailed interaction of the visual cue of relative size with other visual cues, including motion perspective and binocular disparity, remains to be determined. Future models of navigation that take into account such properties of the visual system may allow for more focused, detailed predictions of navigation behavior.

Image matching theories of reorientation are based primarily on evidence from studies of non-human animals, especially rodents [29] and insects [8], [30]. In light of the present findings, experiments using the present displays and methods on other animals will be important to evaluate the differences and similarities across species in the respective roles of 2D-image analysis and 3D depth processing in guiding navigation. We note, however, that the evidence for reorientation by depthless image matching in vertebrate animals also is open to question. First, chicks and fish show patterns of reorientation that are not predictable from an analysis comparing 2D retinal images of the layout [35], [36], [37]. Second, strong behavioral evidence for image-matching in rats comes from trained animals (e.g., [20]), whose disoriented search likely depends not only on automatic processes of reorientation but on learning processes for locating objects relative to proximal landmarks [17]. In fact, evidence for view-matching in chicks comes strictly from their trained navigation behavior using an array of columns [45], the same environmental features that they fail to use in a spontaneous reorientation task [46].

It is also important to consider the relevance of the present findings to the numerous neurophysiological studies assessing changes in the firing fields of spatially selective neurons in the hippocampus and surrounding cortex following the movement of a cue card of distinctive brightness on the border of the navigable space (e.g., [23], [24]). While it is possible that the landmark control over the neuronal firing is indicative of a dissociation between the reorientation of the animal and spatial representations at the neuronal level, a crucial distinction that must be made is that most neurophysiological studies do not disorient the animals and therefore may reflect orientation with an active landmark-anchored path integration system. Supportive evidence for this possibility comes from a study showing that repeated disorientation significantly weakens the control of a cue card over head direction cells and place cells in rats [25] and strengthens the control of the environmental geometry over the head direction cells [47]. Furthermore, in light of the effect of surface brightness on children’s reorientation [31], [32] and the degree of sensitivity to subtle differences in perceived distance in the present study, it remains a question whether a white cue card in an arena made of dark walls subtly perturbs the perceived environmental symmetry.

Despite the ubiquity of reported brightness effects, we have failed, in three experiments testing 80 children, to find evidence that children reorient by differences in surface brightness, either in square or in subtly rectangular rooms, even though our tests used arenas that closely matched those of past studies and methods that yielded positive findings both in those studies and in Experiments 1–3. Why do children reorient by brightness differences in some studies [31], [32] but not others?

One possible reason for the differing findings of these experiments concerns the lighting conditions used in different studies. In one study [31], each of the four walls of the rectangular chamber was illuminated directly. It may be that illuminating the walls directly enhances the salience of their differences in brightness. However, this interpretation does not explain the successful use of surface brightness in other experiments in which surfaces were not directly illuminated [32], or the selective successes and failures of the present experiments. An alternative explanation is that the depth cue of *shading* depends critically on the light source: When the real or perceived source of illumination changes, so do the perceived depth relations within a display (e.g., [40], [48]). If brightness differences influence children’s reorientation by modulating their perception of surface distances, then brighter surfaces will appear closer to the child than darker surfaces only when the room appears to be illuminated by an internal source. In the present experiments, six symmetrically placed fluorescent lights, far above the test array, created diffuse lighting with no clear directional source. Thus, the lighting arrangements used in the published studies of the brightness effect may have created a clearer impression of an internal light source, evoking an impression of relative distance. If this account is correct, then brightness differences, like pattern size differences, may influence reorientation by perturbing the perceived shape of the enclosure. Such a hypothesis could explain why brightness differences influence disoriented animals’ navigation more robustly in otherwise symmetrical environments (e.g., a square or circular array) than in rectangular environments (e.g., [7]), whose shape specifies environmental directions with or without the brightness cue.
